# Dengue and US Military Operations from the Spanish–American War through Today

**DOI:** 10.3201/eid1804.110134

**Published:** 2012-04

**Authors:** Robert V. Gibbons, Matthew Streitz, Tatyana Babina, Jessica R. Fried

**Affiliations:** Armed Forces Research Institute of Medical Science, Bangkok, Thailand (R.V. Gibbons);; Uniformed Services University of the Health Sciences, Bethesda, Maryland, USA (M. Streitz, T. Babina);; Mahidol-Oxford Tropical Medicine Research Unit, Bangkok (J.R. Fried)

**Keywords:** dengue, military personnel, history, fever, viruses, vector-borne infections, United States, Spanish–American War, military operations

## Abstract

Dengue is a major cause of illness among travelers and a threat to military troops operating in areas to which it is endemic. Before and during World War II, dengue frequently occurred in US military personnel in Asia and the South Pacific. From the 1960s into the 1990s, dengue often occurred in US troops in Vietnam, the Philippines, Somalia, and Haiti. We found attack rates as high as 80% and periods of convalescence up to 3-1/2 weeks beyond the acute illness. The increase in dengue throughout the world suggests that it will remain a problem for military personnel until an effective vaccine is licensed.

## Abstract 2

Dengue may remain problematic for military personnel until an effective vaccine is licensed.

Dengue has proven itself a challenge to US military personnel. Even though case-fatality rates are low, dengue can rapidly incapacitate personnel. Dengue caused major illness among US service members stationed in the Philippines beginning after the Spanish–American War, and although not reported in the Iraq and Afghanistan conflicts, it has occurred during many others since that time.

To assess the effect of dengue on US military personnel stationed in dengue-endemic areas, we performed a literature search using “dengue” and “military” (109 titles), “army” (126), “navy” (22), “air force” (7), and “war” (29) and selected articles relevant to the US military. We searched personal files and reviewed military histories and books. References in these publications were reviewed for additional pertinent articles.

Before the Vietnam War, a diagnosis of dengue was usually based on clinical findings, sometimes supplemented by a complete blood count. The clinical diagnosis of dengue, especially in epidemiologically permissive settings of immunologically naive personnel assigned to tropical countries, is relatively accurate. Carefully described outbreaks of dengue in immunologically naive adults are almost pathognomonic. In 2 studies in the Philippines during 1924–1925 (*1*) and 1929–1930 (*2*), patients who had not traveled in dengue-endemic areas before or after the study were experimentally infected with the dengue virus, and clinical dengue developed . More than 40 years later, serologic testing confirmed that the patients in the first study had been infected with dengue virus serotype 1 and those in the second study with serotype 4 (*3**,**4*). In addition, a study from the Vietnam era serologically confirmed 77%–80% of clinically diagnosed dengue (*5*). Characteristics that identify a febrile outbreak as dengue include predominant leukopenia, maculopapular rash, retro-orbital headache, and a relatively long period of incapacitation after defervescence.

The references documented that since the Vietnam War, dengue has been diagnosed by hemagglutinin inhibition, plaque neutralization, complement fixation, and/or virus isolation. In most cases, assays (not sampling) were done after the illness to determine its etiology.

## Before World War II

### Cuba

After the Spanish–American War in 1898, US troops were stationed in Cuba, Puerto Rico, Panama, and the Philippines. In Cuba, troops had widespread and debilitating fevers from typhoid, malaria, and yellow fever, among other illnesses. The principal vector for dengue and yellow fever, *Aedes aegypti* mosquitoes, was common in urban areas. Distinguishing dengue was a lower priority than distinguishing yellow fever and typhoid (*6*). The number of missed dengue diagnoses is unknown. A dengue epidemic in Cuba occurred in 1897, and some researchers have linked troop movements to subsequent outbreaks in Texas and Florida during the ensuing 3 years (*7**,**8*).

Among the second occupation force during the first decade of the 1900s, dengue reportedly occurred without causing any deaths. The most serious health threat throughout the new occupation was typhoid fever, which appeared in localized epidemics, occasionally causing deaths (*9*).

In 1903, with US encouragement, Panama proclaimed independence, and the Hay–Bunau-Varilla Treaty granted rights to the United States in a zone of ≈10 × 50 miles. In 1904, US Navy physicians reported 200 cases of dengue from the Isthmus of Panama (*10*). Exact numbers were not given, but reports noted that “[d]engue has already played an important part in increasing the ratio of sick days among the men stationed in our most recently acquired territory” (*11*).

### Philippines

**Figure 1:**
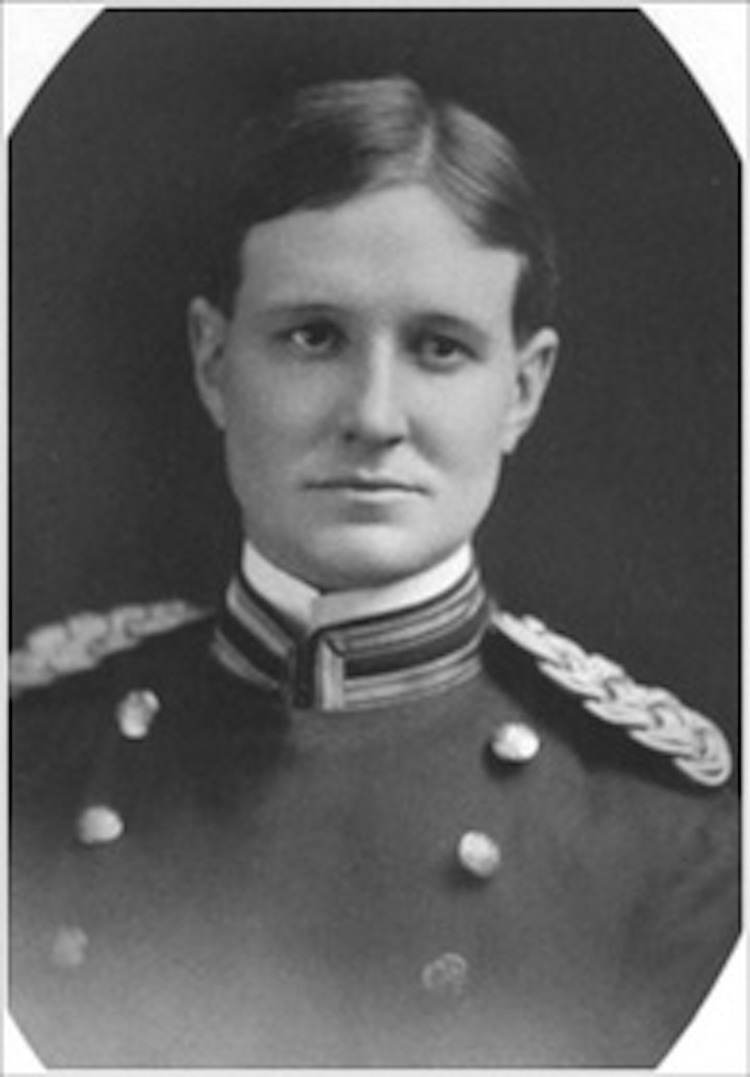
Captain Percy Ashburn.

**Figure 2:**
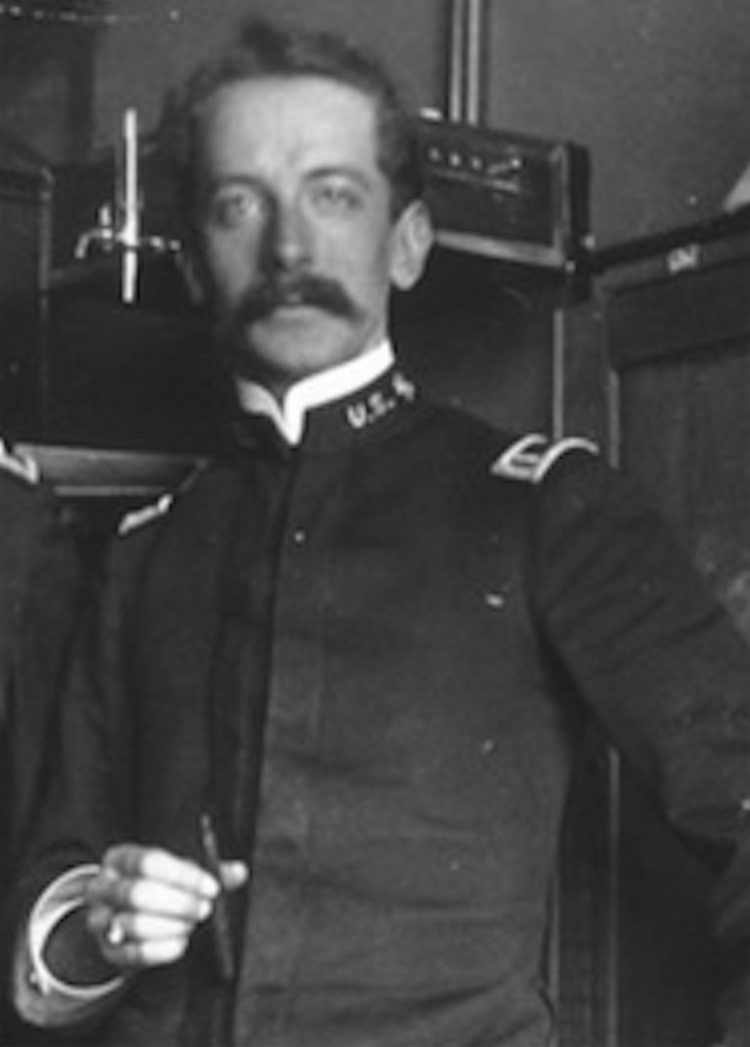
First Lieutenant Charles Craig.

The Army Tropical Disease Board in the Philippines was created in 1898 to investigate a wide variety of health problems that threatened military and civilian populations. According to Brigadier General George H. Toney, dengue caused a “small constant non-effective rate” among the troops (*9*). In 1906, a dengue epidemic swept Fort William McKinley, located on a low site near Manila, and the study of dengue became a priority for members of the Board, including Percy Ashburn (Figure 1) and Charles Craig (Figure 2) (*9**,**12*).

**Figure 3:**
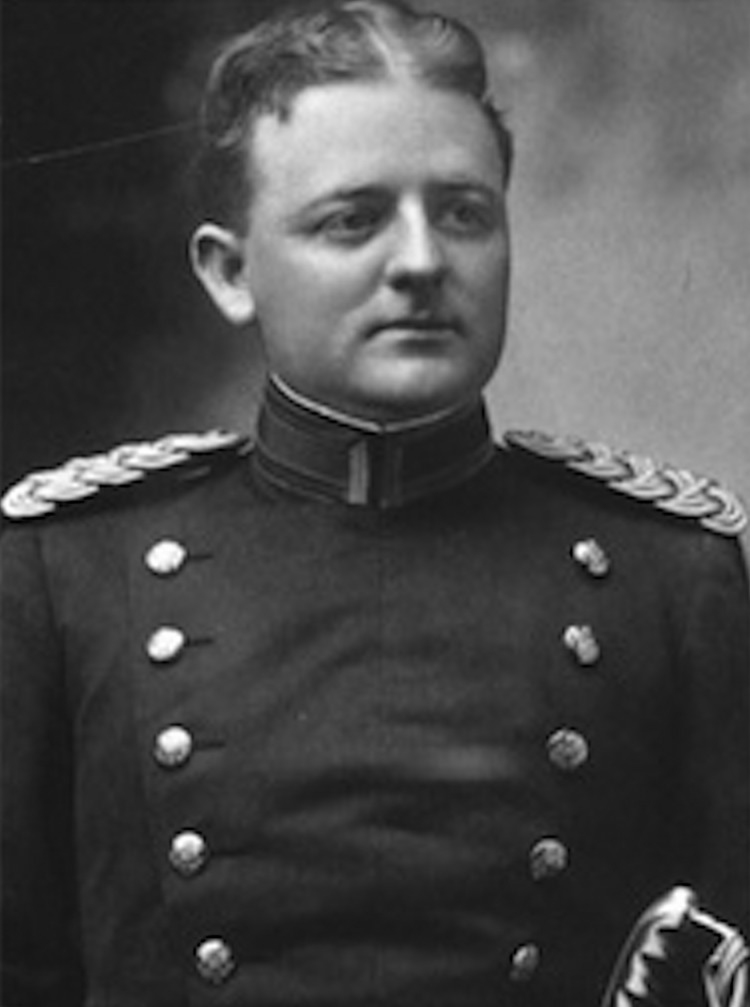
Lieutenant Commander J.F. Siler.

The Philippine tour of duty was usually 2 years, and dengue-naive persons were arriving with each transport of troops. During 1902–1924, hospitalizations for dengue averaged 101 per 1,000 persons per year (range 12–213/1,000/year), and the average hospitalization lasted 7 days (*6*). Lieutenant Colonel J.F. Siler (Figure 3) recognized that the greatest risk was in the Manila urban environment; rates of disease were much lower in remote posts. Approximately 40% of newly arrived troops acquired dengue within 1 year; for 30% (12% of the total), illness recurred during their tour, and for 15% (<2% of the total and most of those staying beyond 2 years), dengue occurred a third time. Siler et al. proposed that these percentages underestimated disease incidence because most of the fevers of short duration (3–6 days) were of unknown cause and could have included dengue. Although rates of illness in general for troops in the Philippines had declined by half during 2 decades, rates for dengue did not appreciably change. Dengue was second only to venereal disease as the most common illness during this period (*1*).

In 1928, Major James Simmons et al. found that annual hospitalizations for dengue per 1,000 troops per year were 6.84 for the entire Army (0.02 for the United States; 0.5 for Panama; and 177 for the Philippines) (*2*). Not only did >98% of cases occur in the Philippines, but also >96% were from Manila and surrounding areas. During 1925–1928, ≈4,000 work days were lost each year to hospitalization for dengue (*2*).

## World War II

**Figure 4:**
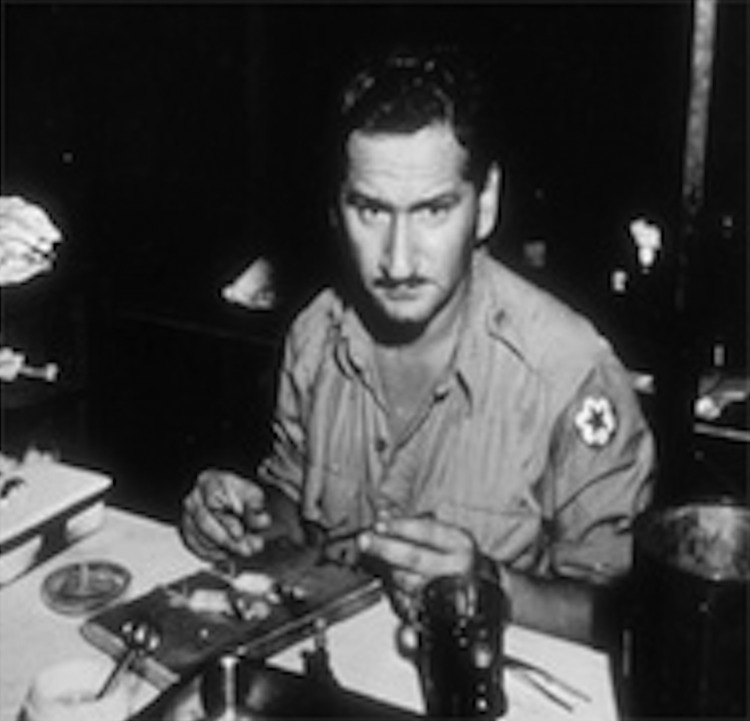
Major Albert Sabin.

During 1942–1945, dengue was diagnosed in only 245 soldiers in Latin America (mostly from the Panama Canal Zone), compared with ≈80,000 who were hospitalized for dengue in the Pacific Theater, in addition to ≈8,000 in the China-Burma-India Theater (*13*). The epidemics engendered continued study of dengue, including Albert Sabin’s research in pursuit of an effective dengue vaccine (Figure 4).

### Australia

A dengue epidemic occurred in 1942 among US personnel stationed in Queensland and the Northern Territory; 80% of service members were affected during a 3-month period (*13*). A subsequent epidemic (463 cases) occurred during January–March 1943 (Table 1). Major Joseph Diasio et al., reporting from an analysis of 100 cases among US service members in Australia, found that the average hospitalization was 7.5 days. Informally, they observed in a small sample that patients needed another 7–10 days to return to full strength (*14*).

**Table 1:** Dengue in US service members during World War II*

Location	Dates	Attack rate, %	No. cases	Maximum no. cases/1,000/y	Reference
North Territory and Queensland, Australia	1942 Mar–May	80	ND	ND	(*13*)
Rockhampton/Brisbane, Australia	1943 Jan–Mar	ND	463	ND	(*14*)
Espiritu Santo, archipelago of New Hebrides (now Vanuatu)	1943 Feb–Aug	25	≈5,000	1,713	(*15*)
New Caledonia	1943 Jan–Aug	ND	ND	645	(*13*)
	1943 Jan–Aug	ND	ND	120	
Hawaii	1943	ND	56	ND	(*16**,**17*)
Gilbert Islands	1944	ND	396	26	(*16*)
New Guinea	1944 Jan–Dec	ND	24,079	198	(*13*)
	1945 Jan–Aug	ND	2,960	31	
Philippines†	1944 Nov–Dec	ND	2,012	49	(*13*)
	1945 Jan–Dec	ND	8926	32	
Saipan, Mariana Islands	1944 Jul–Sep	ND	~20,000	3,560	(*13**,**16*)
China-Burma-India	1943	ND	ND	25	(*13*)
	1944	ND	ND	31	
	1945	ND	ND	8	
Okinawa, Japan	1945 Apr–Aug	ND	≈865	275	(*18*)
Hankow, China	1945 Sep	83	40	ND	(*13*)

*ND, no data. †Reported to have reached 68 cases/1,000 service members/year in the Sixth Army.

### South Pacific

The Malaria and Epidemic Control Board of the South Pacific Area rated dengue second only to malaria as a tropical disease of military importance (*15*). This finding remains unchanged today (*19*). Dengue profoundly affected operations because of the weakness and fatigue that persisted for weeks after the acute phase. Dengue was reported to have caused nearly 1,600 hospitalizations during spring 1942 among Allied prisoners at the Changi Prisoner Camp on Singapore Island (*20*). The US military moved rapidly in the South Pacific to establish military bases without allowing time for precautions and prevention measures to avert the spread of dengue. The military focused on such imperative issues as food, ammunition, construction of defensive positions, and fighting; concern for local diseases, especially nonfatal diseases, was not a priority. The constant traffic of personnel and supplies between islands of the South Pacific contributed to the circulation of dengue by providing susceptible hosts and vector breeding sites.

**Figure 5:**
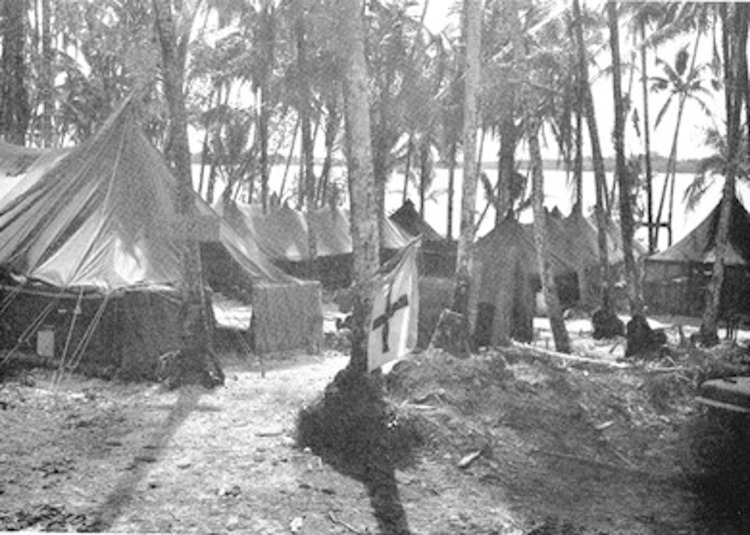
New Georgia Island medical clearing station, Solomon Islands, 1943.

Commander James Sapero and Lieutenant Commander Fred Butler reported “almost all troops” located in Tulagi (Solomon Islands) were affected by dengue shortly after ground action ceased in August 1942. They speculated that the evacuation of infected patients facilitated the spread of dengue in the South Pacific (Figure 5). Within 3 months in the Espiritu Santos area, dengue cases caused illness rates to increase from 12% to 40% (*21*); affected service members were absent from strenuous duty for at least 2 weeks (*22*). One publication reports an epidemic in the archipelago of New Hebrides (now Vanuatu) on the island of Espiritu Santo in 1943. The epidemic began in February, peaked in April and subsided in August; 25% of the base strength (≈5,000 personnel) was affected, with a maximum of 1,713 cases per 1,000 persons per year (*13**,**23*). The epidemic also affected New Caledonia but to a lesser extent (Table 1) (*13*).

Zeligs et al. reported that in July 1943, four members of an aviation unit flew from 1 unidentified island to another (*24*). Shortly after their arrival at the second island, dengue was diagnosed in all 4. At the same time, a dengue epidemic was identified on the first island. Traffic between the 2 islands could not be stopped because of the support required for combat operations, and the influx of personnel to the first island continued. To prevent the spread of disease, strict measures were enforced. Infected personnel were placed in an isolated camp, and the remaining servicemen were closely monitored for signs and symptoms (*24*). This transmission phenomenon was seen elsewhere. One author, reporting on an advanced base in Melanesia, wrote of dengue being brought by patients from neighboring islands, which resulted in 80,000 sick days and attack rates as high as 12% (*25*). In addition, in 1944, a total of 396 dengue-infected military personnel from the Gilbert Islands were evacuated to hospitals on Oahu, Hawaii (*16*).

Another author, writing of the epidemic in Marine and Navy personnel in the South Pacific, estimated that one third were affected and that a “large group were hospitalized.” He noted, “The acute attack of dengue lasted for about 8 days, the convalescent period often ran into weeks before the patient could return to his previous type of duty” (*26*). One article noted that ≈2% of patients had pain so severe that they required morphine for relief (*27*).

Others reported 1,200 cases of dengue in March and April 1943 in Army troops on an unidentified island (*28*). Observers of this outbreak reported that temporary immunity existed for 5–10 months after an episode of dengue; after several attacks, more lasting immunity existed. The convalescent period was generally 2–3.5 weeks but even longer for older patients. The Thirteenth Air Force, operating in the South Pacific, reported that during March, 49 days were lost per 100 flying officers (*15*).

Severe outbreaks of dengue were reported on Saipan, an island in the Marianas. The first occurred in July 1944 in the Marshall Islands, when dengue was diagnosed in 744 persons, most of whom were on Saipan. The disease reportedly was much more clinically severe than it had been in 1943 (*16*). In August, 300 cases per 1,000 persons per year occurred and rapidly jumped to 3,500 per 1,000 per year by September 1944. With the arrival of DDT in September, the Army enacted a plan to control mosquitoes in the area. DDT and kerosene were sprayed from airplanes during September 13–22, 1944. Ten days of spraying seemed extremely effective; the attack rate decreased to 182 cases per 1,000 persons per year by October (*13**,**16*).

Dengue cases among the staff of 2 major hospitals located on Saipan, the 148th General Hospital and the 176th Station Hospital, demonstrated the effectiveness of vector control through spraying. The former hospital arrived on August, 10, 1944, and the latter ≈6 weeks later. Spraying began on September 13, ≈1 week before the 176th Station Hospital opened. In the interim, the 148th General Hospital saw infection rates for staff as high as 47% (252 personnel), amounting to a rate of 3,500 cases per 1,000 persons per year. In contrast, the 176th Station Hospital experienced no dengue cases among its staff, probably because of improved vector control. Of 4,624 troops who arrived during September 17–30, a total of 41 (0.9%) cases occurred (232 cases/1,000 persons/year) (*16**,**23*).

### Hawaii

After an absence of >30 years, dengue was reintroduced to Hawaii in July 1943 when commercial airline pilots carried the disease from the South Pacific to Honolulu. A dengue outbreak first appeared along Waikiki beach, resulting in the August 8 declaration of the area as off limits to the troops. Local authorities created a squad to go door to door inspecting premises and providing instructions and education to the public about preventing dengue (*17*,*23*).

Because of the strategic importance of the area and the role already played by dengue in combat operations, the Army designated soldiers to perform inspections along with the civilian squad. Travel was restricted among the Hawaiian Islands. Despite these measures, dengue cases in Waikiki increased. To prevent further spread, all premises in Waikiki were sprayed, and more soldiers were assigned to the inspection squad to help with mosquito elimination. Eventually, mosquito control was extended citywide, led by the US Public Health Service; most labor was provided by an Army medical service company (*13*). Additional areas were declared off limits to the troops (*17*). By June 1944, cases in 1,500 civilians and 56 military personnel had been reported (*16**,**17*).

### Okinawa

The Army in Okinawa experienced a dengue outbreak during spring and summer 1945. Incidence peaked among members of an infantry unit at 275 cases per 1,000 persons per year in July. The authors noted 161 cases in a field hospital, 704 in a clearing station, and numerous others in various Army and Navy medical facilities. The average hospital stay was ≈7 days. None of the hospitalized patients required evacuation, and all returned to active duty (*18*).

### New Guinea and Philippines

**Figure 6:**
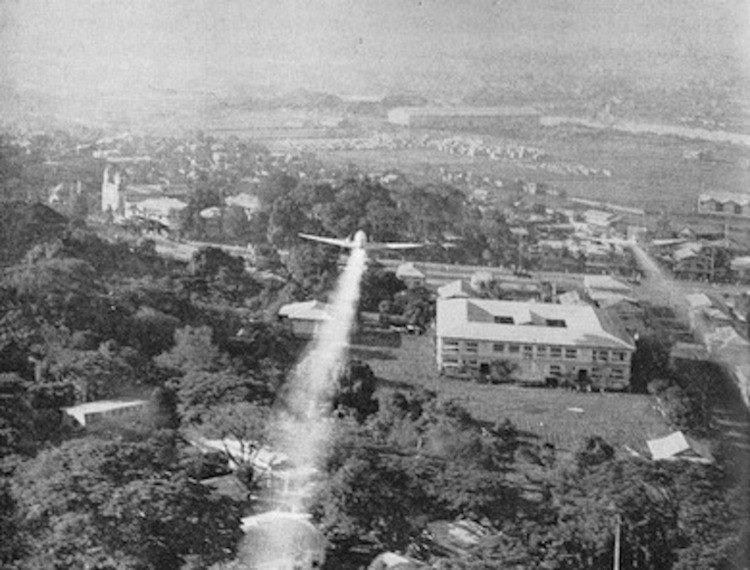
Airplane spraying of DDT over Manila, the Philippines, 1945.

From the start of operations in New Guinea, dengue was a major cause of loss of troop strength. Statistics available for 1944–1945 indicate ≈27,000 cases; epidemics were reported in the Hollandia and Biak areas. By contrast, in the Philippines, dengue cases occurred only sporadically and without epidemic proportions, perhaps because of the extensive use of DDT in populated areas on Luzon from the beginning of the reoccupation (Figure 6) (*13*).

### China-Burma-India Theatre

Most reported dengue cases in the China-Burma-India Theater occurred in Calcutta, reaching rates of 31 cases per 1,000 persons per year in 1944. In addition, the famed Merrill’s Marauders reportedly were adversely affected by dengue. In September 1945, a dengue outbreak occurred in Hankow, China, which was reported to have affected 80% of the population, including Japanese personnel. Of the first 48 US troops to occupy the airport in Hankow, dengue developed in 40 within 5–10 days. The city area was deemed off limits, and a unit was ordered into the area for mosquito control (*13*).

## Vietnam War

At the end of World War II, 2 dengue serotypes were discovered (*29**,**30*). During the decade leading up to the Vietnam War, 2 additional serotypes were identified, and dengue was found to cause a more severe illness, dengue hemorrhagic fever (*31*).

In 1964, an outbreak of dengue occurred in Ubol, Thailand, among US and Royal Australian Air Forces (Table 2). Of 294 men, dengue was confirmed for 16%–19% (*5*). A study conducted during 4 months in 1966 at the 93rd Evacuation Hospital in Long Binh, South Vietnam, evaluated 110 cases of fever of unknown origin (FUO, i.e., fever and a negative malaria smear in patients whose illness remained undiagnosed 24 hours after hospitalization). Of these, dengue was diagnosed in 31 (28%) and was the most prevalent disease causing FUOs. The researchers concluded that dengue was acquired within the urban setting of the base camp (*32*). Another study of FUOs (excluding malaria diagnosed during the first 72 hours after hospitalization) was conducted for 4 months during 1966–1967 at the Eighth Field Hospital in the semimountainous central coastlands of Vietnam. Ten (11%) of 94 cases were dengue (*33*). Nine patients came from more inhabited rather than rural regions. A third study of FUOs among 87 soldiers deployed to the rural Mekong River Delta in 1967 found that 3% of cases were caused by dengue (*34*).

**Table 2:** Dengue in US service members during the Vietnam War

Location or source of samples*	Dates	Dengue cases among fevers of unknown origin, %	Total no. fevers of unknown origin	Reference
Ubol, Thailand	1964 May–Aug	77–80	69*	(*5*)
Vietnam				
93rd Evacuation Hospital, Long Binh	1966 Apr–Aug	28	110	(*32*)
8th Field Hospital, Nha Trang	1967 Oct–Feb	11	94	(*33*)
Dong Tam, Mekong Delta	1967 Jun–Dec	3	87	(*34**,**35*)
I Corps	1967 Feb–Sep	3	295	(*35*)
12th US Air Force Hospital	1968 Jul–Jun	5	306	(*35**,**36*)
12th US Air Force Hospital	1969	10	1,256	(*35**,**36*)

*Attack rate in this study was 16%–19%.

During May 1965–April 1966, the average monthly incidence of dengue in US Army personnel in Vietnam was 3.5 cases per 1,000 troops (range 1.2–6.7/1,000) (*38*). As shown in FUOs studies, dengue was underreported because of lack of laboratory capabilities. FUOs during the same period ranged from 9.1 to 101.0 cases per 1,000 persons per month (average 55.2/1,000/month); dengue constituted a substantial fraction. In 1967, the monthly incidence of dengue was 57–87 cases per 1,000 troops (*39*) (average 75/1,000) (*40*). A 1-year study from the 12th US Air Force Hospital at Cam Ranh Bay during 1967–1968 found that dengue caused 15 (5%) of 306 FUOs (*36*). In a 2-year study of servicemen residing separately from native populations and with 4 days of FUO in 6 Navy-Marine hospitals, 5 (0.6%) of 377 cases resulted from dengue (*37*). A summary of FUOs from Vietnam in 1969 found that 10% were caused by dengue (*35*). Unlike during World War II, dengue never reached major epidemic proportions among the troops in Vietnam. Nevertheless, a variety of studies attributed 3.4%–28% of “fever of undetermined origin” cases to dengue in service members who had had contact with the local population. Using these percentages with FUO numbers from the same period, we can calculate a monthly dengue incidence of 2–15 cases per 1,000 persons during 1965–1966 and 3–21 per 1,000 during 1967. Days lost because of FUOs averaged 225,000 per year during 1967–1970 (reference *41* in Technical Appendix).

Although the more severe dengue hemorrhagic fever occurred among Vietnamese children, no cases were diagnosed in the troops. Most troops were unlikely to have been exposed to a second dengue virus infection, which predisposes them to more severe disease.

## After the Vietnam War

### Philippines

In 1984, Clark Air Base, north of Manila, had a population of ≈10,000 personnel. During June–September 1984, a total of 42 confirmed cases and 9 probable cases of dengue occurred. Of these, 35 occurred in military personnel and 25 (71%) persons were hospitalized. Hospitalization ranged from 3 to 11 days (average 5.9 days), and patients reported not being fit for duty for 3 to 18 days (average 14.6 days). One person was admitted to the intensive care unit and shock subsequently developed. By the end of September 1984, the vector populations were markedly reduced by an extensive education program and mosquito elimination strategies (*4*; *42* in Technical Appendix).

### Somalia

More than ≈30,000 US troops went to Somalia as part of Operation Restore Hope during 1992–3. Of 289 patients hospitalized with fever during that operation, 129 (45%) did not have an immediately identified cause of illness. Of the 96 tested for dengue, 59 (61%) had positive results; dengue thus accounted for at least 20% of hospitalizations. Illnesses remained unspecified for 24%; many might have been dengue (*43* in Technical Appendix).

An additional serologic study was performed on a military unit that had 26 (5%) members discharged from the hospital with unspecified febrile illness; dengue was confirmed for 17 (65%) (13 by virus isolation and 4 by IgM). A subsequent serosurvey showed that an additional 27 members of the unit had seroconverted to dengue virus; 16 had a febrile illness, 4 had nonfebrile illness, and 7 were asymptomatic. Thus, up to 7.5% (17 + 16 + 4 = 37 of 493) of the unit had dengue (*43* in Technical Appendix). Some cases that remained unspecified were possibly dengue with waning IgM. In another study of patients consecutively hospitalized with fever, dengue viruses were isolated from 14 (17%) of 81, and serologic test results were positive for 15 (18%) of 84 (*44* in Technical Appendix). In addition, dengue was confirmed in journalists and relief workers seeking care at US military field hospitals (*45* in Technical Appendix). According to these studies of Operation Restore Hope, dengue accounted for 7%–21% of illness.

### Haiti

In September 1994, ≈20,000 US military personnel deployed to Haiti as part of Operation Uphold Democracy. During the first 6 weeks, 30 (29%) of 103 patients hospitalized with febrile illness had confirmed dengue (22 virus isolation, 8 IgM); dengue was excluded for 40 (39%) cases, and cause was undetermined in 31 (30%). Patients came from urban and rural environments (*46* in Technical Appendix). These numbers did not include outpatients. During the follow-up United Nations mission in Haiti, dengue was diagnosed in 79 (32%) of 249 soldiers and civilians who had fever and sought care at the 86th Combat Support Hospital. The actual numbers were probably much higher because only IgM testing was conducted (*47* in Technical Appendix). In another report from the United Nations Mission in Haiti, dengue was confirmed in 233 (56%) of 414 suspected cases (*48* in Technical Appendix).

## The Present

Many US military operations involve small numbers of personnel in diverse locations. During October 2008–October 2010, dengue developed in at least 9 Special Forces soldiers. Recently, a report was published about a Special Forces soldier deployed in South America who became ill with dengue and required evacuation from a rural setting (*49* in Technical Appendix); another report described a Marine who required hospitalization during deployment to the Philippines (*50* in Technical Appendix). During 1999–2008, a total of 97 dengue cases (45 in the Army) (7 cases per million person-years) were reported among the active-duty personnel of the US Department of Defense (*51* in Technical Appendix). A recent seroprevalence study of 500 samples from US Army Special Forces soldiers during 2006–2008 found antibodies against dengue in 11% (*52* in Technical Appendix). No cases have been reported in the Iraq or Afghanistan conflicts.

## The Future

Dengue has substantially weakened US military operations and reduced troop strength since the Spanish–American War. Recognizing these facts, the Military Infectious Disease Research Program and the Medical Research and Materiel Command have supported dengue vaccine research. A recent quantitative algorithm for prioritizing infectious disease threats to the US military rated dengue third behind malaria and bacterial diarrhea (*53* in Technical Appendix). Historically, the military significance of dengue has probably been underestimated (*54*,*55* in Technical Appendix). As US deployments around the globe continue, dengue prevention is needed for service members and other persons in dengue-endemic regions. Dengue vaccine development, despite many unique challenges, is moving forward and is the best hope for protection against dengue (*56* in Technical Appendix).

## Supplementary Material

Technical Appendix Supplementary References.
