# Supplementary material for: Dengue and US Military Operations from the Spanish–American War through Today
Source: Emerg Infect Dis. 2012 Apr;18(4):623–30. doi: 10.3201/eid1804.110134 (PMC3309667; doi:10.3201/eid1804.110134)
Supplement: Technical Appendix — Supplementary References. [file 11-0134-Techapp_2p.pdf]

Article DOI: http://dx.doi.org/10.3201/eid1804.110134

Dengue and US Military Personnel from Spanish–American War through Today

Technical Appendix

Supplementary References

41. Neel S. Health of the Command. In: Medical support of the US Army in Vietnam 1965–1970. Washington (DC): Department of the Army; 1973. p. 32–48.

42. Hayes CG, O'Rourke TF, Fogelman V, Leavengood DD, Crow G, Albersmeyer MM. Dengue fever in American military personnel in the Philippines: clinical observations on hospitalized patients during a 1984 epidemic. Southeast Asian J Trop Med Public Health. 1989;20:1–8. PubMed

43. Sharp TW, Wallace MR, Hayes CG, Sanchez JL, DeFraites RF, Arthur RR, et al. Dengue fever in US troops during Operation Restore Hope, Somalia, 1992–1993. Am J Trop Med Hyg. 1995;53:89–94. PubMed

44. Kanesa-thasan N, Iacono-Connors L, Magill A, Smoak B, Vaughn D, Dubois D, et al. Dengue serotypes 2 and 3 in US forces in Somalia. Lancet. 1994;343:678. PubMed http://dx.doi.org/10.1016/S0140-6736(94)92678-6

45. Sharp TW, DeFraites RF, Thornton SA, Burans JP, Wallace MR. Illness in journalists and relief workers involved in international humanitarian assistance efforts in Somalia, 1992–93. J Travel Med. 1995;2:70–6. PubMed http://dx.doi.org/10.1111/j.1708-8305.1995.tb00630.x

46. Trofa AF, DeFraites RF, Smoak BL, Kanesa-thasan N, King AD, Burrous JM, et al. Dengue fever in US military personnel in Haiti. JAMA. 1997;277:1546–8. PubMed http://dx.doi.org/10.1001/jama.1997.03540430058033

47. Gambel JM, Drabick JJ, Swalko MA, Henchal EA, Rossi CA, Martinez-Lopez L. Dengue among United Nations mission in Haiti personnel, 1995: implications for preventive medicine. Mil Med. 1999;164:300–2. PubMed

48. Rossi CA, Drabick JJ, Gambel JM, Sun W, Lewis TE, Henchal EA. Laboratory diagnosis of acute dengue fever during the United Nations Mission in Haiti, 1995–1996. Am J Trop Med Hyg. 1998;59:275–8. PubMed

49. Taylor SF, Taylor CL. Dengue fever with hemorrhagic features in a special forces soldier. Prehosp Emerg Care. 2006;10:494–501. PubMed http://dx.doi.org/10.1080/10903120600725983

50. Trayers FJ III, Simon J, Praske SP, Christopher KL. Fever, headache, and myalgias after deployment to the Philippines. Mil Med. 2008;173:1188–93. PubMed

51. US Armed Forces. Defense medical epidemiology database [cited 2009 Mar 20]. http://www.afhsc.mil/aboutDmed

52. Caci JB, Lyons A, Tack D. Seroprevalence of dengue fever in US Army Special Operations forces—initial results and the way forward. Annual Meeting of the American Society of Tropical Medicine and Hygiene; 2010 Nov 3–7; Atlanta. Abstract 90.

53. Burnette WN, Hoke CH Jr, Scovill J, Clark K, Abrams J, Kitchen LW, et al. Infectious diseases investment decision evaluation algorithm: a quantitative algorithm for prioritization of naturally occurring infectious disease threats to the US military. Mil Med. 2008;173:174–81. PubMed

54. Kuno G. Research on dengue and dengue-like illness in East Asia and the Western Pacific during the first half of the 20th century. Rev Med Virol. 2007;17:327–41. PubMed http://dx.doi.org/10.1002/rmv.545

55. Smallman-Raynor MR, Cliff AD. Impact of infectious diseases on war. Infect Dis Clin North Am. 2004;18:341–68. PubMed http://dx.doi.org/10.1016/j.idc.2004.01.009

56. Thomas SJ. The necessity and quandaries of dengue vaccine development. J Infect Dis. 2011;203:299–303. PubMed http://dx.doi.org/10.1093/infdis/jiq060
